# Supplementary material for: Optimal minimal residual disease threshold in pediatric acute myeloid leukemia: A retrospective cohort study based on the TARGET database
Source: PLoS Med. 2026 May 8;23(5):e1005088. doi: 10.1371/journal.pmed.1005088 (PMC13155632; doi:10.1371/journal.pmed.1005088)
Supplement: S1 Code — (ZIP) [file pmed.1005088.s002.zip › S2 code/PROJ8_15_tbl1/PROJ8_15_tbl1_0.pdf]

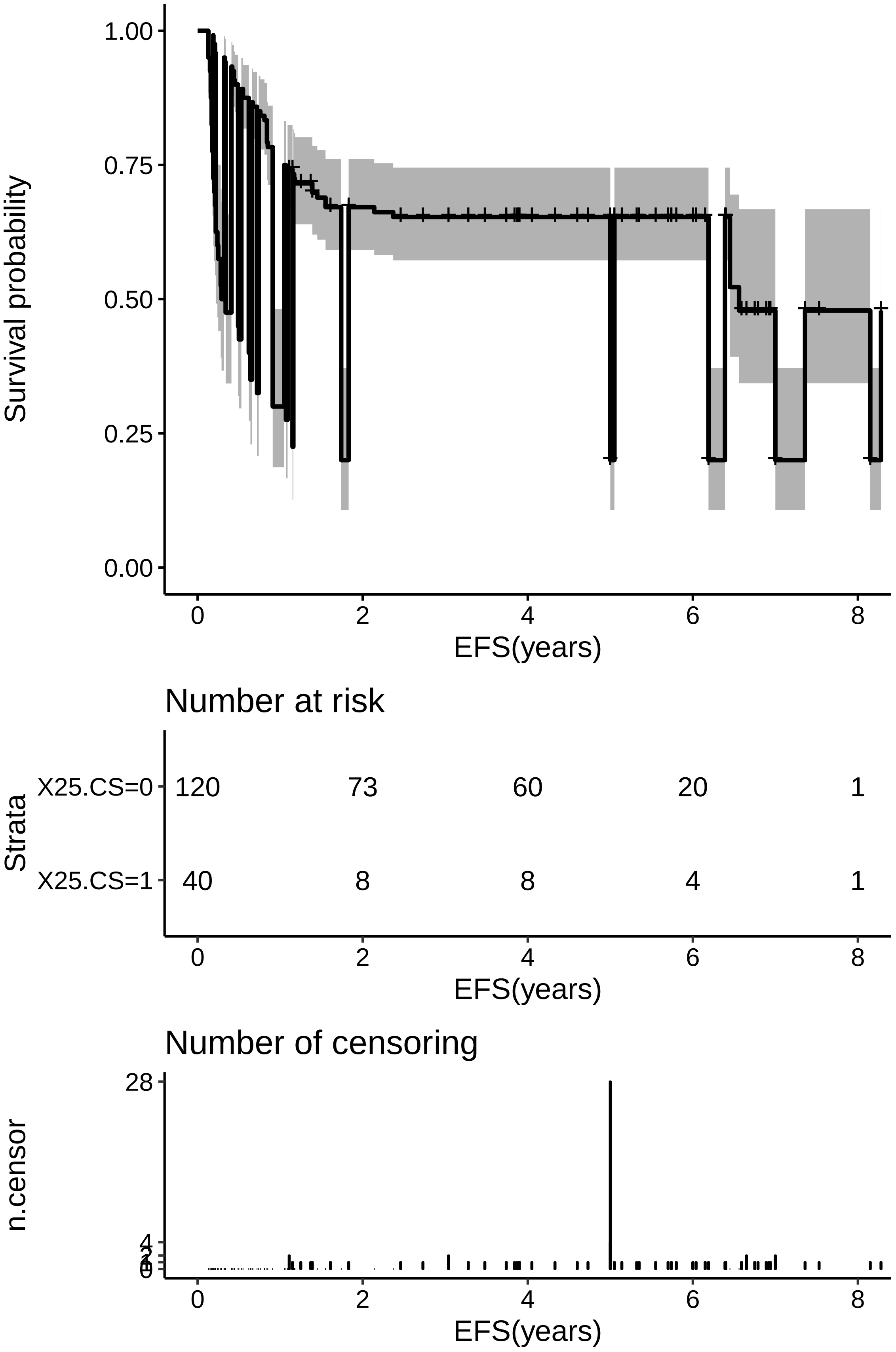

Survival probability
1.00
0.75
0.50
0.25
0.00
0 2 4 6 8
EFS(years)
Number at risk
Strata
X25.CS=0 120 73 60 20 1
X25.CS=1 40 8 8 4 1
0 2 4 6 8
EFS(years)
Number of censoring
n.censor
28
4
0
0 2 4 6 8
EFS(years)
